# A Look at the Interconnection of Dimensions of Knowledge in Physical Education Teacher Training in Chile

**DOI:** 10.3390/ijerph20043249

**Published:** 2023-02-13

**Authors:** Sebastián Peña-Troncoso, Sergio Toro-Arévalo, Javier Vega-Ramírez, Francisco Gallardo-Fuentes, José María Pazos-Couto

**Affiliations:** 1 Institute of Education Sciences, Faculty of Philosophy and Humanities, Universidad Austral de Chile, Valdivia 5091000, Chile; 2 Faculty of Education and Culture, Universidad SEK, Santiago 7520317, Chile; 3 School of Physical Education, Faculty of Education, Pontificia Universidad Católica de Chile, Santiago 8331150, Chile; 4 School of Physical Education, Faculty of Philosophy and Education, Pontificia Universidad Católica de Valparaíso, Quillota 2260000, Chile; 5 Department of Physical Activity Sciences, Universidad de Los Lagos, Santiago 8370341, Chile; 6 Faculty of Education and Sport Sciences, Universidad de Vigo, 36310 Pontevedra, Spain

**Keywords:** attitudinal assessment, preservice teacher education, physical education

## Abstract

The massive fragmentation of knowledge that exists in the current field of physical education enables us to research pedagogical and disciplinary aspects in the educational processes of teachers in training, as this has significant implications for future educational practices. This study proposes to assess the dimensions of knowledge (conceptual, procedural and attitudinal) that stem from the learnings that emerge in physical education teacher training in terms of the disciplinary standards proposed by the Chilean Education Ministry for the Preservice Teacher Education. The study methodology was descriptive and inferential, and the cohort was cross-sectional. A total of 750 fourth- and fifth-year students in training from 13 Chilean universities participated. Of these, 619 subjects were considered: 54.6% (338) men and 45.4% (281) women between the ages of 21 and 25. The questionnaire used for data collection was the “Questionnaire on Conceptual, Procedural and Attitudinal Learning in Preservice Teacher Education in Physical Education” (CACPA-FIDEF), prepared as part of Fondecyt project No. 11190537. The main results indicate that there are no statistically significant differences in the three dimensions in terms of students’ sex and type of schooling, with *p* values > 0.05. In conclusion, the study observed a weak conceptual management of the discipline in future teachers, revealing once again the need to seek out didactic alternatives that enable teachers in training to understand the importance of the conceptual dimension in their learning and teaching processes.

## 1. Introduction

Preservice Teacher Education (PTE) is a space–time characterized by complexity [1], as it constitutes an educational process constructed at the intersection of different variables that condition and enable it, where the central theme is the confluence of the triad of fields of knowledge, namely disciplinary, pedagogical and practical education [2]. With this understanding, there is a multiplicity of elements that, when combined, demand a series of skills from teachers in training, such as the deployment of capacities developed throughout their lives and during their university education that make it possible for them to demonstrate and respond to the challenges of this process [3]. Therefore, PTE responds to life processes, to public policies and to lines of epistemic education that support certain visions of education in general and teacher training in particular. Currently, Chile has a Professional Teacher Development System regulated by Law No. 20,903 [4], which makes it mandatory for universities that offer pedagogical education in Chile to have Pedagogical and Disciplinary Standards (EPD in Spanish), explicitly establishing that teacher education should enhance certain knowledge, skills and attitudes [5,6] (Mineduc-Chile (2021) [6] defines knowledge as conceptual and declarative knowledge and distinctions that explain, describe or conceptualize a field of social knowledge. Skills are understood as procedures or standardized or emergent ways of acting that are specific to a field of knowledge or social practice. Attitudes are understood as evaluative dispositions and ethical commitment to fields of knowledge or social practices recognized as relevant to the school curriculum).

Compliance with this law assumes an educational minimum required to establish a professional identity and capacity that guarantees coherence in training and subsequent performance, regardless of the place and institution at which a teacher is trained. From this perspective, in physical education teacher training in Chile, the challenge has been constant and enormous efforts have been made in an attempt to achieve a comprehensive education that enables future teachers to implement practices with a critical sense, which have an impact on the daily life of students based on their own interests and contexts, leaving behind ways of understanding teacher training from more technical paradigms typical of the period after the 1990s. However, the didactic deployment of physical education teachers in the context of the school system continues to be characterized by a strong activist, morpho-functional and technical emphasis, privileging the procedural, with a high focus on biomedical aspects [7]. In this way, it does not consider what is required by public policy, nor does it provide a comprehensive understanding of the discipline. Therefore, in our opinion, it becomes an obligation and a necessity to overcome this technical paradigm and focus efforts on the training of teachers with a critical and reflective sense, which allows for a comprehensive and non-fragmented training of knowledge [8,9]. 

This conception of the discipline for biomedical and technical purposes has led to a fragmentation, not only in macro-curricular terms in PTE for physical education, but also at micro-curricular levels, specifically from the dimensions of knowledge (knowledge, skills and attitudes). This situation, which in turn is passed on in school education by giving priority to physical tests, controls and tests of execution, pushes to the side or omits conceptual and attitudinal training [9,10]. 

The erroneous understanding held by the populace regarding physical education [11] has led both PTE for physical education and the school context to focus the discipline on sports and physical performance and on the encouragement and development of athletic talent to such an extreme that it asks teachers to improve the performance results of the student body by forming selective sports teams in traditional sports, thereby adopting processes of selectivity and exclusion. 

This morphofunctional outlook, typical of a dualistic and mechanical–functional perspective and ontology toward physical education [12,13], focused on technified learning [14] and highlighting performance assessment in determining physical condition [15], will keep us far from a comprehensive and systemic education of teachers in training. As long as we fail to recognize and operationalize the discipline as an educational act whose development is based on, and intended for, relative autonomy through different displays of human motor skills, we will be out of place, reproducing only instructional processes rather than educational ones, and with it, physical education will be left out of the historical, social and personal processes in terms of global approaches to human knowledge and existence. 

From this point of view, the great challenge for the different universities is still to comprehensively train Physical Education teachers, moving from a reductionist fragmentation of knowledge to a systemic and multidimensional understanding of it, both at the curricular level but also fundamentally in the didactic proposals that they build, thus moving towards inter- and transdisciplinary dialogues in formal school education [16,17], recovering the prominence of the processes of socialization, context and interest of children and young people [18]. There is still a significant gap between teacher training institutions and the school system [19], highlighting that fragmentation is not only epistemic, but also political and ontological, so it is urgent to move towards a systemic and complex approach [7,8,20] which allows us to understand and promote the constitutive and confluent nature of the procedural, conceptual and attitudinal dimensions, and the importance of deepening and/or broadening them.

## 2. Teacher Professional Development in Physical Education Teacher Training in Chile

Law No. 20,930, in force since 2016, establishes a Teacher Professional Development System and the obligation to have pedagogical and disciplinary standards for programs and degrees that impart pedagogies in Chile. This expresses a political and epistemic intention to recognize the crucial role of teacher education and its responsibility to ensure that the country prepares future education professionals with the necessary skills to support a universe of increasingly diverse and heterogeneous students in the achievement of increasingly complex and challenging learning. This law tasks the Chilean Education Ministry (MINEDUC) with developing performance standards. These, in conjunction with the specific standards of each pedagogical degree program, make up what are known as standards of the teaching profession.

The milestone that kicked off this policy of standards for pedagogy degree programs was a document published in 2011 by the Center for Improvement, Experimentation and Pedagogical Research (Centro de Perfeccionamiento, Experimentación e Investigaciones Pedagógicas, CPEIP), which set the guiding standards for graduates of elementary education pedagogy programs, and later in 2014 for specialized pedagogies, including physical education [4]. From that moment, these standards have been a fundamental pillar in the education of teachers in different universities throughout the country. In 2021, new pedagogical and disciplinary standards were published [5] and will be in force as of 2023. These standards aim to ensure that future teachers are able to demonstrate a command of the knowledge of their discipline, the epistemology and history from which it is constituted, and specific didactic knowledge for teaching this. What is relevant about these standards is that they were prepared with a much more situated and systemic perspective, collecting the fundamentals of motor skills from a conception situated in learning.

In this sense, the standards set the challenge to move past the conception of learning as a reproduction of the existing culture and to make way for a pedagogy that legitimizes experiential learning lived, contextualized and in interaction with the reality where we are tasked with practicing the profession. Out of this emerges a notion that each child and young person is the protagonist of their own story, with full dignity, from a perspective of critique and possibility. In short, from this construction of disciplinary standards linked to a humanized and social approach to the educational realm, the perspective of teaching in the subject of a pedagogy of being is grounded in the meaning of corporeality and motor skills in the existence and development of children and young people, responding to the question regarding the specific good or meaning that the discipline provides to society [5] (pp. 77–78).

These new standards make it possible to observe the discipline from a very systemic and integrative paradigm, allowing the different Physical Education teacher training courses to become more flexible and contextualized from the possibilities provided by their contexts. This gives rise to a unitary and complex perspective of the discipline that is articulated by both the General Education Act and the Teacher Professional Development Act [4], emphasizing the comprehensive training of the country’s citizens in the context of a complex and knowledge-based society. Therefore, it is important to train teachers with a critical and emancipatory sense, capable of developing new skills and competences in different contexts, so that young people can be effective workers and citizens of the knowledge society [21].

Law 20,930 establishes a mechanism for identifying what teachers in training have learned; specifically, two diagnostic assessments that must be undertaken by pedagogy students: The first is applied by the university at the beginning of the program and the second by the CPEIP 12 months before the end of the program. The second assessment is based on the pedagogical and disciplinary standards for PTE for the purpose of generating information on the pedagogical and disciplinary knowledge obtained by students during their university education [5]. This second assessment and its correction guidelines are not known in advance. The university is only notified of the results of the assessment applied for each standard. It is important to note that this assessment is a requirement for the certification of future teachers and is also considered an important input for the revision and improvement of university training programs and actions designed to support teachers who must undertake the CPEIP assessment.

### From the Interconnection of Contents to the Multidimensional Condition of Knowing

Although we normally refer to types of content (conceptual, attitudinal and procedural), from our perspective, we understand knowledge as a multidimensional process, since each knowledge requires an act, an understanding and a value or ethical commitment based on the context in which everyone is situated [22]. This perspective makes it possible to understand learning as a triple construction, sustained in an operation-sense and concept at the same time. This three-dimensionality is not necessarily expressed and generated in the same way at all times, but rather it varies depending on the culture and evolution of the learner; nonetheless, there is clarity and consensus that these occur simultaneously. We recognize and understand that it is not possible to fragment the dimensions of knowledge in educational terms. However, it is important to understand in didactic and curricular terms how these dimensions are already configured in the learning–teaching context [23], i.e., procedural, attitudinal and conceptual. 

The conceptual dimension is identified with “knowing” information that we can remember exactly as it was memorized [24,25]. The main characteristic is that the student can verbalize and communicate it [26] or that it is formed through language [27]. The value of considering the conceptual dimension is that it allows students to understand and communicate what they have learned, rather than just operating as mere agents regurgitating an action determined by the teacher [8].

The second dimension, “attitudinal”, considers the elements that are not directly measurable in people, but observable in their way or style of acting, taking into account that they are affected by beliefs and feelings that predispose them to express agreement or disagreement towards a given construct [28] and that they therefore imply a personal or contextual ethical commitment. This is how attitudes reflect the value placed by subjects on other people, ideas or things [29]. This personal opinion may be negative, positive or neutral [30] and is directly influenced by the quality of human interactions, which takes on significant relevance since it represents the existential and moral anchoring of what each trainee learns through life. As an internal dimension that is not directly observable, attitude is learned or, some would say, constructed by people throughout their lives [31,32] and presents three components: affection, behavioral tendency and cognition [28]. These components refer to the feelings and emotions (affection), intentions or dispositions toward action (behavior) and thoughts and beliefs (cognition) of the person towards the construct of interest [29]. 

The third dimension, “procedural”, refers to “how”, the way of doing or behavior that implies specific knowledge or learning and is connected to actions that put the understanding and application of a learned skill into practice [33,34]. According to Monereo [24], this type of content can be closed (logarithmic) or open (heuristic). The relevant aspect is that these are ways of operating that are observable and show evidence of a certain knowledge: ways of operating that characterize and identify knowledge. For example, running in general, as knowledge, is different than running in the context of track and field, and this in turn can vary depending on the type of test, i.e., speed, resistance or mixed. We understand, then, that each action contemplates the three dimensions that, together as a whole, express the understanding, value placement and operation implied by any act of knowing.

From this perspective, considering the aforementioned information, the study aimed to assess PTE for Physical Education teachers in Chile based on the three dimensions of knowledge (conceptual, procedural and attitudinal) based on the disciplinary standards issued by the Chilean Education Ministry through a valid and reliable instrument.

## 3. Methodology

### 3.1. Method and Sampling 

The research that we present corresponds to a descriptive and inferential study with a cross-sectional cohort. Questionnaire items and scores were prepared and validated with a sample of university students. Students participated from a total of 13 Chilean universities. To determine the number of participants, a non-probabilistic sampling was applied, where participants were chosen non-randomly and for convenience. A pilot test was initially carried out with 237 students in their final year of the physical education pedagogy program and the final test was later applied to 750 students. Of these, 619 were considered valid (338 men and 281 women, aged between 21 and 25 years).

### 3.2. Instrument 

The instrument used to collect the information was the “Questionnaire on Conceptual, Procedural and Attitudinal Learning in Preservice Teacher Education in Physical Education” (CACPA-FIDEFIS), prepared after reviewing the Disciplinary Standards for Physical Education Pedagogies [6] based on a coverage matrix and different discussion groups with teachers and students in PTE. The research team decided to prepare a series of questions that respond to disciplinary standards, placing a clear emphasis on the three dimensions of knowing—knowledge (conceptual), skills (procedural) and dispositions (attitudinal)—that students in training must understand within their academic training. The instrument questions in the conceptual and procedural dimensions were raised in the form of statements (proposals) and questions, with four responses provided for each. However, after carrying out the first pilot questionnaire, in an effort to improve the discrimination indexes of each question and its answers, the research team decided to transform the statements into questions, also modifying the four response options. For the attitudinal dimension, the questions were prepared on a scale where 1 = never; 2 = rarely; 3 = frequently; 4 = always.

### 3.3. Instrument Validity and Reliability

The expert assessment technique was used to determine the validity of the instrument. This procedure evaluates the degree to which a test adequately represents what has been done [35,36]. This included the participation of 8 researchers/teachers with many years of experience in physical education teacher training. Three variables were used to assess content validity: (a) Degree of adequacy of the questions in the questionnaire (on a conceptual level); (b) Degree of relevance to the study object; (c) Degree of adequacy on a level of definition and understanding. After the expert assessment and subsequent analysis of the instrument, the team decided to eliminate 10 out of a total of 66 items as they had scored below 7 points based on the criteria established by Bulger and Housner [37]. The second version of the instrument therefore included 56 items.

A pilot test was subsequently carried out with students (*n* = 237) with similar characteristics to those who participated in the study. A verification was run on the percentage of discrimination for each answer and the difficulties in relation to the wording, adequacy and specific terminology of the various questions and answers. The team eliminated 8 questions, given that the percentage of answers in the alternatives were very heterogeneous. This resulted in the third version of the questionnaire, reducing the total to 48 items.

To determine the internal and temporal reliability of the questionnaire, the team decided to recode the questions in the conceptual and procedural dimension, transforming them into dichotomous ones (1 = correct; 2 = incorrect). The Kuder–Richarson Formula 21 (KR-21) test designed for dichotomous variables [38] was used to calculate internal reliability, obtaining a coefficient of 0.57, a value that allows us to estimate a moderate internal consistency of the instrument [39]. Cronbach’s alpha was used for the value placement or attitudinal dimension, obtaining a coefficient of 0.87, a value that allows us to estimate a high degree of reliability in this dimension.

To achieve optimal levels of temporal reliability of the questionnaire, the test–retest technique was used with a sample of 35 students through the intraclass correlation coefficient (ICC), which allowed us to assess concordance between both measures. A coefficient of 0.48 was obtained for the conceptual and procedural dimension and a coefficient of 0.74 for the attitudinal dimension, showing a highly significant relationship (*p* < 0.01). Although the ICC value that defines a satisfactory reliability of the instrument is arbitrary and varies depending on the use it is given, in general, ICC values below 0.4 are low in terms of reliability, values between 0.4 and 0.75 are fair to good, and values above 0.75 are excellent in terms of reliability [40]. It is important to note that the sample of participants showed the same characteristics as the students surveyed. Following the proposal [41], both measurements were taken under identical circumstances, separated over the span of one week.

Finally, the construct validity of the instrument was determined through the use of exploratory factor analysis (EFA). EFA was performed on the instrument at two stages, mainly because the constructs for the conceptual and procedural dimensions were recoded into dichotomous responses; however, the construct for the attitudinal dimension was composed through a Likert scale (1–4). The first EFA was performed through a tetrachoric correlation [42] as the variables are dichotomous, and the second EFA through a polychoric correlation. The Kaiser–Meyer–Olkin (KMO) test used to measure the sampling adequacy of the first exploratory factor analysis (conceptual and procedural) confirmed an adequacy of 0.62; therefore, the idea is acceptable. Bartlett’s test of sphericity was 0.00. Kaiser–Meyer–Olkin (KMO) for the second exploratory factor analysis (attitudinal) confirmed the analysis with an adequacy of 0.88, and Bartlett’s test of sphericity was 0.00. Both analyses confirmed the assumption that there is an internal correlation in the answers obtained after the application of the instrument. The extraction of common factors from the items were analyzed using Robust Unweighted Least Squares (RULS) as the extraction method. As a procedure to explore the number of factors, the optimal implementation of parallel analysis (PA) [43] was used on the polychoric matrix. A Normalized Varimax rotation was also used. The first analysis was grouped into two factors: Factor 1—conceptual; Factor 2—procedural, which explains the 0.28 % of total variation (Table 1). The second analysis grouped three factors: Factor 3—Motor skills; Factor 4—Assessment; Factor 5—Planning (Table 2), explaining the 0.44% total variance. Three items were excluded in the first exploratory factorial analysis (item 14, item 23 and item 26), which registered below 0.3 or difficult on the difficulty index [44]. The instrument consisted of a final total of 45 items. 

In the attitudinal dimension, due to the nature of the questions and the scoring assigned to these when analyzing the results, items 26, 29, 30, 33 and 34 were inverted.

### 3.4. Procedure

Due to the health scenario that has affected society as a whole, the questionnaire was conducted online through the Questionpro platform (https://www.questionpro.com/a/TakeSurvey?tt=C20EsU4oDK8%3D) (accessed on 7 February 2023). Prior to the assessments, meetings were held with the program boards of directors and the National Accreditation Council of Physical Education (*Consejo de Acreditación Nacional de Educación Física*, CANEF), who helped manage the relationships with the universities. The nature of the research was explained to the boards and the respective permits were obtained. Because the survey was online, students were required to provide an informed consent before beginning the questionnaire. The study was approved by the Ethics Committee of the Universidad Austral de Chile. All students participated voluntarily and with respect for the Helsinki agreement on research ethics in reference to research with human beings [45].

### 3.5. Data Analysis

A descriptive analysis of central tendency (mean) and dispersion (standard deviation) was carried out, as well as an inferential analysis through the Student-t method, to determine if there were differences based on sex. An ANOVA was also applied for the type of schooling variable. The discrimination and reliability analyses were performed with SPSS statistical software version 28 and EFAs were performed with Factor Analysis 10.4 software [46]. Significance was established for *p* < 0.05.

## 4. Results

Table 3 shows a low conceptual command of motor skills, negatively highlighting that 86.4% of the teachers in training demonstrate that they are unclear about the epistemic foundation of motor skills, 81.4% are unaware of muscle strength training, 72.5% of analytical training and 65.3% do not recognize the physical qualities that degenerate with age. However, among the positive results, 52% say they know the curricular bases of 11th and 12th grade, 55.7% recognize the characteristics of a qualitative assessment and 53.3% say they know the foundation of a cumulative assessment. These results provide evidence of the low conceptual background in the discipline, mainly regarding motor skills and types of training.

From the procedural dimension, three fundamental aspects stand out in Table 4, with over 50% giving the correct responses. A total of 61.7% of teachers in training are aware of how to proceed if a student feels unwell on a field trip (example: trekking), 53.6% know how to carry out a self-assessment and 51.9% know the elements that they consider necessary before implementing the class. These results are overshadowed, mainly by the 87.1% who do not know how to proceed with a peer assessment, the 67% who do not understand how to activate basic, specific and specialized motor skills with children, and finally, the 66.4% who do not know how to incorporate activities that strengthen indigenous cultural experiences in Chile. These results reveal that there is still controversy regarding the role of the teacher in promoting spaces for peer assessment among students and the activities that are conducive to activating basic, specific and specialized motor skills.

Table 5 shows the results of the attitudinal dimension, which is made up of three large categories. The first is related to the value placed on the formative assessment by the students in training. Among the most relevant results, 79.8% consider the personal abilities of students to be important in the assessment, 85.6% consider the process important and, finally, 74.2% place relevant value on the final achievement obtained by their students. However, when considering the criteria “frequently” and “always”, 79.3% still consider the physical performance of the students to be relevant and 73.9% the physical tests. These value placements ratify the strong paradigmatic value placed on measurements and results that still prevails in physical education.

The second category relates to motor skills and their manifestations, observing among students in training a high value placed on four aspects that they consider important in terms of motor skills: (a) first aspect: 81.4% value the students’ health; (b) second aspect: 77.3% value cognitive development; (c) third aspect: 77.2% value actions and (d) fourth aspect: 75.3% value games. However, when considering the criteria “frequently” and “always”, 64.7% of the teachers in training value sports training, revealing once again that sports-oriented training continues to be a priority for many universities and their PTE curricula for physical education.

Finally, the planning category shows that, when planning classes, teachers in training place high value on the context in which the class is planned (89.2%), the capacities of the students (83.5%), the materials they have (84.8%) and the prior knowledge that each student brings (78.2%). However, once again there is an explicit emphasis on results and grades when planning, without placing much importance on each student’s processes. If we consider the criteria “frequently” and “always”, we can see that there is a high value placed on class results, with 94.8%, and grades, with 79%.

Table 6 presents the results in terms of the mean score and standard deviation obtained through total responses based on each dimension, disaggregated for the students’ sex and type of schooling. For the conceptual dimension, the maximum score is 10 and the minimum is 1; for the procedural dimension, the maximum score is 6 and the minimum is 1. Finally for the attitudinal dimension, the maximum score is 4 and the minimum is 1. Considering the results of each dimension, no statistically significant differences are observed in the three dimensions in terms of sex and type of schooling, with *p* values > 0.05. However, women are observed to have a higher command of the discipline. For the type of schooling variable, the conceptual and attitudinal dimensions show that students who graduated from municipal schools have a better understanding of the discipline, and the procedural dimension shows a better decision-making capacity among students who graduated from subsidized schools.

## 5. Discussion

Different empirical evidence can be seen at a school level [47,48,49,50], which ratifies the importance of the conceptual dimension in physical education. As a discipline, for many years, its main focus has been on motor action resulting from “knowing how to do”. However, when observing the study results for the conceptual dimension, we can infer that in physical education teacher training, this trend remains, revealing the low conceptual background that exists in the discipline, mainly from the understanding of motor skills and the types of training that are implemented there.

Considering the above, it is precisely because of this hegemonic conception of the discipline focused on procedural skills that the focus of teachers and researchers is always on motor practice, oriented towards “sports performance” [51,52] as well as “learning time” [53,54], while at the same time, as seen in the studies based on anthropometric measurements, there is a marked focus on being overweight and obesity [55,56,57,58]. 

Despite the large amount of research focused on procedural aspects, the study results are not very encouraging. The results observed in Table 6 show that the students in training recognize that they do not understand the procedures in terms of different didactic practices—for example, a peer assessment, which for many continues to be a grading process [59]. Therefore, as long as we are unable to understand assessment and grading as two clearly different albeit related processes, it will be impossible to change our professional practice. However, this repeats despite studies that show the futility of this grade in the teaching and learning processes [60]. 

In attitudinal terms, the results of this dimension show a high value placed on the three categories of the construct (motor skills, assessment and planning), and the latter shows us that students in training value the planning process based on the contents of the study program, i.e., the curricular bases of the discipline, which was already expressed in the study by Almonacid et al. [61]. For future teachers, it is relevant to have a structured planning within the framework of the curricular bases, which makes it possible to guarantee quality teaching, and it is necessary to consider the entry behavior of the students, their abilities and interests, and the diversity of learning levels [62]. In terms of assessment, the cognitive attitude revealed in this category is very favorable, considering this type of assessment to be relevant in the learning and teaching processes. This coincides with the results reported by Gallardo-Fuentes et al. [63], which reveal a high value placed on the perception of formative and shared assessment by students in preservice education training to be physical education teachers. Finally, from the area of motor skills, the value placed is also very positive in cognitive terms. However, there is still confusion regarding the epistemological foundation of motor skills and their manifestations, associating and limiting motor skills to movement [64].

## 6. Conclusions

The study of the dimensions of knowledge in PTE for physical education can place tension on the fragmented outlook of the discipline. From this perspective, it is necessary to generate a new outlook around the learning achieved by students in training to be PE teachers, which one way or another transcends into the education of schoolchildren. In general, a weak conceptual management of the discipline was observed in future teachers, revealing the need to seek didactic alternatives that allow them to understand the importance of the conceptual dimension in their learning and teaching processes. Regarding the procedural dimension, we can conclude that there is a certain degree of confusion regarding different didactic procedures of the discipline. Although some procedural actions are highlighted in field trips and self-assessments, the concern is focused on peer assessment processes and activation of basic, specific and specialized motor skills. 

From the attitudinal dimension, we can conclude that teachers in training place high cognitive value on the context in which the classes are planned, on the formative assessment as an identification process of the personal capacities of students and on motor skills, through different manifestations in health and games.

Finally, in terms of global results (Table 6), we can infer that there is a poor understanding of the conceptual and procedural dimension and that there are no statistically significant differences in the three dimensions based on sex and type of schooling, with *p* values > 0.05.

The training of physical education teachers continues to be a great challenge in PTE. From this perspective, a quantitative and qualitative improvement is necessary in conceptual, procedural and attitudinal training, seeking a balance in understanding, action and value placement in all the learning environments across university education experiences. This emphasizes the importance of generating spaces for dialogue and reflection regarding learning, projecting a classroom setting that lets them recognize the value of the discipline from a multidimensional approach. Therefore, for greater epistemic and disciplinary coherence, it is time to reformulate the disciplinary categories, recognizing that PTE is becoming more systemic and complex every day, both in terms of public policy and the experience and development of learning.

## Figures and Tables

**Table 1:** Matrix of rotated components.

ITEMS	Factor 1	Factor 2
Item 9	0.515	
Item 10	0.528	
Item 11	0.357	
Item 12	0.440	
Item 13	0.359	
Item 15	0.483	
Item 16	0.383	
Item 17	0.532	
Item 18	0.561	
Item 19	0.469	
Item 20		0.618
Item 21		0.545
Item 22		0.603
Item 24		0.319
Item 25		0.468
Item 27		0.581

Note: Item 14, item 23 and item 26 were excluded due to a difficulty index below 0.3. Factor 1 = Conceptual Dimension. Factor 2 = Procedural Dimension.

**Table 2:** Exploratory factorial analysis of the attitudinal dimension.

ITEMS	Factor 3	Factor 4	Factor 5
Item 28	0.657		
Item 29	0.593		
Item 30	0.716		
Item 31	0.619		
Item 32	0.426		
Item 33	0.550		
Item 34		0.777	
Item 35		0.798	
Item 36		0.701	
Item 37		0.711	
Item 38		0.759	
Item 39		0.566	
Item 40		0.741	
Item 41		0.807	
Item 42			0.591
Item 43			0.631
Item 44			0.665
Item 45			0.765
Item 46			0.629
Item 47			0.778
Item 48			0.741

Factor 3 = Motor skills. Factor 4 = Assessment. Factor 5 = Planning.

**Table 3:** Descriptive results for the conceptual dimension.

Conceptual Dimension	Responses	
	Frequency/% Correct	Frequency/% Incorrect
9: From the paradigm of complexity, motor skills are:	84/13.6%	535/86.4%
10: Pre-sports games help to incorporate into sports:	254/41.0%	365/59.0%
11: When applying exercises in class, the teacher must be conscious of the demands on students in accordance with their capacities and characteristics. What is the main training principle that should be considered?	264/42.6%	355/57.4%
12: The essential recommendation to build a camp with the students is:	288/46.5%	331/53.5%
13: Training muscle strength is characterized by:	115/18.6%	504/81.4%
15: When planning training analytically, this must consider:	170/27.5%	449/72.5%
16: The degenerative physical quality that reduces with age is:	215/34.7%	404/65.3%
17: The new curricular bases for 11th and 12th grade emphasize the development of:	322/52.0%	297/48.0%
18: Assessment is a qualitative expression to focus learnings through:	345/55.7%	274/44.3%
19: The purpose of the cumulative assessment is focused on:	330/53.3%	289/46.7%

**Table 4:** Descriptive results for the procedural dimension.

Procedural Dimension	Responses	
	Frequency/% Correct	Frequency/% Incorrect
20: From the didactics of physical education, you need to work with the conditional (physical) capacities of 9th grade students. What elements do you consider essential before implementing your class?	321/51.9%	298/48.1%
21: You need to design and implement different activities that favor the understanding of basic, specific and specialized motor skills in 12-year-old girls and boys. How would you do this?	204/33.0%	415/67.0%
22: You need to incorporate human motor skills to expand the indigenous cultural experiences of students in our country. What would you choose?	208/33.6%	411/66.4%
24: You need your students to complete a self-assessment on the team sports unit. How would you go about this?	332/53.6%	287/46.4%
25: You need your students to complete a peer assessment on the corporal expression unit. How would you conduct this assessment?	80/12.9%	539/87.1%
27: You are trekking a volcano with 11th grade students and on the ascent, one of the students doesn’t feel well and can’t go on. What do you do?	382/61.7%	237/38.3%

**Table 5:** Descriptive results for the attitudinal dimension.

Attitudinal Dimension				
	Never	Rarely	Frequently	Always
**When I think of the formative assessment**				
28: I think of the personal capacities of my students	6.0%	1.8%	17.8%	79.8%
29: I think of the physical performance of my students	1.8%	18.9%	49.9%	29.4%
30: I think of my students’ processes	0.0%	8.0%	13.6%	85.6%
31: I think of the physical tests that I can apply	2.7%	23.4%	53.2%	20.7%
32: I think of my students’ final achievements	1.3%	7.3%	17.3%	74.2%
33: I think of my students’ grades	7.8%	60.1%	20.4%	11.8%
**When I think about motor skills and their manifestations**				
34: I think of my students’ corporeity	1.5%	4.5%	26.7%	67.4%
35: I think of my students’ emotional development	5.0%	6.8%	23.7%	69.0%
36: I think of sports training	4.8%	30.5%	51.1%	13.6%
37: I think of my students’ health	8.0%	1.5%	16.3%	81.4%
38: I think of games	5.0%	2.7%	21.5%	75.3%
39: I think of sports	1.9%	12.8%	61.9%	23.4%
40: I think of my students’ cognitive development	5.0%	2.3%	19.9%	77.3%
41: I think of my students’ actions	2.0%	1.5%	21.2%	77.2%
**When I plan my classes**				
42: I think of the class results	3.0%	4.8%	26.8%	68.0%
43: I think of the materials I have	3.0%	3.2%	11.6%	84.8%
44: I think of the study program contents	0.0%	5.0%	26.3%	68.7%
45: I think of the context where I will hold my class	0.0%	1.8%	9.0%	89.2%
46: I think of how I would rate the class	2.3%	18.7%	53.6%	25.4%
47: I think of the personal capacities of my students	0.0%	1.5%	15.0%	83.5%
48: I think of my students’ prior knowledge	0.0%	2.4%	19.4%	78.2%

**Table 6:** Results for the conceptual, procedural and attitudinal dimensions disaggregated for the students’ sex and type of schooling.

	Sex			Types of Schooling			
Dimensions	Men	Women		Municipal	Subsidized	Private	
	Mean (SD)	Mean (SD)	Value *p*	Mean (SD)	Mean (SD)	Mean (SD)	Value *p*
Conceptual	3.60 (1.58)	3.70 (1.58)	0.25	3.72 (1.54)	3.58 (1.62)	3.69 (1.51)	0.63
Procedural	2.39 (1.19)	2.47 (1.21)	0.21	2.43 (1.19)	2.46 (1.19)	2.20 (1.27)	0.35
Attitudinal	2.99 (0.18)	3.00 (0.16)	0.19	3.00 (0.16)	2.99 (0.17)	2.99 (0.18)	0.89

Note: Value *p* = Conceptual Dimension: (Means range from 1 to 10). Procedural Dimension: (Means range from 1 to 6). Attitudinal Dimension: (Means range from 1 to 4).

## Data Availability

Not applicable.
